# Psychometric Properties of the Life Events Checklist-Korean Version

**DOI:** 10.4306/pi.2008.5.3.163

**Published:** 2008-09-30

**Authors:** Hwallip Bae, Daeho Kim, Herry Koh, Yangsuk Kim, June Sung Park

**Affiliations:** 1 Department of Neuropsychiatry, College of Medicine, Hanyang University, Seoul, Korea.; 2 Traumatic Stress Clinic, Department of Psychiatry, Hanyang University Guri Hospital, Guri, Korea.; 3 Focus Research, Co., Ltd., Seoul, Korea.

**Keywords:** Life Events Checklist, Reliability, Validity, Trauma, Stressful Events, Factor Analysis

## Abstract

**Objective:**

The Life Events Checklist is a brief screening instrument that is used for potentially traumatic events such as accidents, disasters, sexual or physical assaults, or combat-related exposures. The original English version was recently tested for reliability and validity and it showed good psychometric properties, and so its use is recommended for the assessment of trauma.

**Methods:**

This study investigated the reliability and validity of a Life Events Checklist-Korean version in 157 consecutive psychiatric outpatients at a university-affiliated teaching hospital. The questionnaire also included the Impact of Events Scale-Revised (IES-R), the Beck Depression Inventory (BDI), and the State and Trait Anxiety Inventory (STAI). Correlation and principal component analyses were conducted.

**Results:**

The four week test-retest reliability was good and the internal consistency was acceptable. In addition, the number of traumatic events was significantly correlated with the posttraumatic depressive and anxiety symptoms, which demonstrated the convergent validity of the scale. Additionally, exploratory factor analysis identified a six-factor structure that explained 57.2% of the total variance.

**Conclusion:**

These findings support the reliability and validity of the Life Events Checklist-Korean version.

## Introduction

The majority of people experience traumatic events in their lives. In the United States, it is estimated that 56-90% of the adult population experience at least one traumatic event in their lifetime.^1^-^3^ Likewise, one study on the Korean general population^4^ demonstrated a similar rate of 79% among the adults living in an urban area, and this supports the cross-cultural generalizability of traumatic experience.

Not all the people who experienced traumatic events will later develop a posttraumatic stress disorder (PTSD), a psychiatric condition that involves the trauma-related reexperience, intrusion, avoidance, and hyperarousal.^5^ Among those who experience a traumatic event about 15-25% will go on to develop PTSD.^6^ It should also be noted that other psychopathologies along with PTSD may develop after traumatic events. These conditions include, but are not confined to, major depressive disorder, substance use disorder, and other anxiety disorders.^7^,^8^

To be diagnosed with PTSD according to the Diagnostic and Statistical Manual of Mental Disorders, Fourth Edition (DSM-IV),^5^ a person must first be exposed to (i.e., experience, witness, or be confronted by) potentially traumatic events (PTEs), (criterion A1) and then that person reacts to the event with fear, horror, or helplessness (criterion A2). Thus, screening and identifying the PTEs that patients experience are important prerequisites for establishing the diagnosis of PTSD. However, the literature indicates that among the areas of PTSD assessment, relatively less attention has been paid to the traumatic events (criterion A) than to the symptomatology (criteria B to D).

To date, several scales have been used in clinical and research settings to assess general traumatic events. A recent survey of 227 trauma specialists revealed a dozen adult self-report instruments that are used for this purpose.^9^ However, only a few of these instruments, such as the Stressful Life Events Screening Questionnaire^10^ and the Traumatic Life Events Questionnaire,^11^ have undergone extensive testing for determining their psychometric soundness. The Life Events Checklist (LEC) was recently added to the list after Gray et al.^12^ showed it had the adequate test-retest reliability and good convergent validity.

The LEC is the most widely used adult self-report tools for general PTEs^9^ and it is routinely administered before a structured interview with the Clinician-administered PTSD Scale (CAPS), which is the "Gold Standard" tool for diagnosing PTSD.^13^ In fact, the LEC was developed concurrently with and is embedded within the CAPS. This self-report checklist is used to assess exposure to PTEs before the structured interview.^14^

The LEC is composed of 17 items and each item represents the domain of PTEs from natural disasters to other stressful events. A unique feature of the scale is that the LEC uses five nominal levels of responses: 'happened to me', 'witnessed it', 'learned about it', 'not sure', and 'does not apply'. As the DSM-IV A1 criteria include witnessing and confronting the events in addition to personally experiencing the events, the LEC is advantageous in that it elicits more responses than the other PTE measures, and these responses may that otherwise be overlooked.

Although most attention has been directed to the psychometry of the CAPS, the LEC has recently been proven to have sound psychometric properties.^12^ Likewise, the Korean version of the CAPS showed excellent reliability and high validity^15^; however, the psychometric properties of the LEC have not yet been studied.

This study evaluated the psychometric properties that is, the reliability and the validity of the LEC-Korean version (LEC-K). The factorial validity of the LEC-K was also investigated and this has not yet been reported on in the literature.

## Methods

### Subjects

The data was gathered from 157 consecutive new psychiatric patients (first come, first served) that visited the Outpatient Department of Psychiatry, Hanyang University Guri Hospital in Guri over a one-year period.

All the new patients at the psychiatric outpatient department and who were given a DSM-IV diagnosis^5^ by the attending staff psychiatrists were included in this study. Those patients who had not completed elementary school, as well as those who were judged not capable of completing questionnaires or giving a written informed consent, were excluded. Patients with mental retardation, neurological or cognitive impartment, or severe disorganization were also excluded. The initial screening identified 194 patients who satisfied the study criteria. However, thirty-seven patients (19.1%) refused to participate in the study, and so 157 patients were finally enrolled.

### Procedure

On the first visit, while waiting for the formal consultation, a staff nurse contacted the screened patients to describe the study and to obtain the written informed consent. Those who consented were given a questionnaire set; this was composed of the LEC,^14^ the Impact of Events Scale-Revised (IES-R),^16^ the Beck Depression Inventory (BDI),^17^ and the State and Trait Anxiety Inventory (STAI).^18^ Cross-cultural validation and reliability data are available for the IES-R,^19^ BDI,^20^ and STAI.^21^

Additionally, the clinical and socio-demographic information was obtained from the patients and their medical records. This study was approved by the institutional research ethics board at Hanyang Unviersity Guri Hospital.

### Measures

The LEC has 17 domains of potentially traumatic events (PTEs), and the responses to these items include experiencing, witnessing, and learning about it. This format may enhance responses for PTEs, but in turn, 'witnessing' and 'learning about it' could be confusing to some respondents. For this reason, our study analyzed the responses as a dichotomy, i.e., experience vs. no experience. Thus, witnessing and learning about the incident were both regarded as no experience. The two exceptions were items 14 and 15, which specifically ask about witnessing the events. Therefore, witnessing these items was regarded as experiencing the event.

The IES-R^16^ is a 22-item questionnaire that measures the symptoms of PTSD, which is a representative psychiatric illness that develops after the experience of the traumatic events. The BDI^17^ is a widely used self-rating tool that measures depressive symptomatology. The scale has 21 items and the respondents are asked about various depressive symptoms over the previous week. The STAI^18^ has two separate components: the State Anxiety Inventory (SAI) and the Trait Anxiety Inventory (TAI), and both are made up of 20 items. The SAI measures how a person feels at the present time while the TAI assesses a person's general disposition for anxiety.

### Statistical analysis

The four week test-retest reliability was assessed with a non-random partial convenient sample (n=35) and Cohen's kappa calculation.^22^ The internal consistency of the LEC was evaluated using Cronbach alpha. The correlation between the total number of PTEs endorsed and the psychological impact, the current depression, and the anxiety was sought for determining convergent validity. Further correlation with the demographic factors was assessed for determining the discriminant validity.

For assessing the factorial validity, an exploratory principle component analysis (PCA) with Varimax rotation was conducted. The number of factors was determined by the size of eigenvalues and the variance that was explained by each factor. All the data analyses were conducted using the Statistical Package for the Social Sciences (SPSS) 12.0 for Windows.

## Results

### Subjects

The diagnostic distribution of the participants (n=157) included anxiety disorder (n=71, 45.2%), major depressive disorder (n=35, 22.3%), adjustment disorder (n=17, 10.8%) and others (n=34, 21.7%). The subjects were predominantly women (58.0%) and married people (58.6%). Most had completed high school or they had a higher education (82.6%), and they were employed or they were homemakers (75.2%). The mean age was 34.1 (SD=10.7) years.

### Reliability

The four week test-retest reliability of the LEC-K varied with each item (Table 1). An outstanding Cohen's kappa (above 0.08) was found for motor vehicle accident (Item 3), physical assault (Item 6), and sexual assault (Item 8). Less than moderate kappa (below 0.04) was found for life-threatening injury/illness (Item 12), sudden unexpected death of someone close (Item 15), and caused a serious injury/death of another person (Item 16). The kappa value was not calculated for the four items with too few responses i.e., exposure to toxic substance (Item 5), combat (Item 10), captivity (Item 11), and witness a violent death (Item 14). The mean kappa value for the test items was 0.619. The internal consistency of 17 items was shown by a Cronbach alpha value of 0.667.

### Convergent and discriminant validity

The number of endorsed PTEs on the LEC was significantly correlated with the scores of the IES-R (r=0.329, p<0.001), the BDI (r=0.285, p<0.001), the SAI (r=0.190, p=0.017), and the TAI (r=0.182, p=0.022). However, this was not correlated with status of religion (Spearman's rho=0.024, p=0.770), the marital status (Spearman's rho=0.071, p=0.381), or the level of education (Spearman's rho=-0.066, p=0.415).

### Factorial validity

Exploratory PCA with Varimax rotation of the sample (n=157) identified six factors that explained 57.2% of the total variance: Factor 1 (Physical assault/others), Factor 2 (Accident/injury), Factor 3 (Natural disaster/witnessing death), Factor 4 (Sexual abuse), Factor 5 (Criminal assault), and Factor 6 (Man-made disaster)(Table 2).

## Discussion

In this study, the LEC-K displayed good reliability and validity, which supports the use of this scale for screening PTEs. Of the 13 items for which a kappa value could be calculated, 10 items were above the moderate level (0.04). This finding is consistent with the results of the original version,^12^ including a similar rank order when sorted by the kappa value (e.g., sexual assault, physical assault and motor vehicle accident had the highest kappa values in both studies) and a similar mean value was found for all the items (0.62 in this study vs. 0.61 in the previous study). High test-retest reliability has also been noted in the previous reports that were concerned with sexual or physical assault, and it has been suggested that these emotionally arousing events tend to amplify the memory.^23^

The item, 'life-threatening injury/illness' approached the standard level (0.39-0.40). However, two items did not meet the standard kappa value of 0.04: sudden, unexpected death of someone close (0.36) and caused serious injury/death of another (0.37). A possible explanation for the former would be the ambiguity in defining 'someone close', and the low basal rates of experience may explain the latter. Given the fact that the original previous study sought to obtain one week temporal stability, the four week test-retest reliability in this study could be more acceptable. However, the previous studies measured test-retest reliability of PTEs at the longer intervals (e.g., more than a year), and these previous studies reported inconsistency of at least one traumatic events in 64-88% of the cases.^24^-^27^

Additionally, this study also examined the internal consistency. The Cronbach alpha of 0.67 was above the lenient level of 0.60, but it was below the generally accepted limit (0.70). Additionally, some researchers have criticize the internal consistency analysis of PTE measurement; it has often been proposed that PTE exposure is not a unidimensional construct.^28^

The convergent validity of the LEC-K was demonstrated with the significant correlation between the number of items endorsed and the measures of general symptoms (depression and anxiety) and the trauma-specific psychopathology (psychological distress from a traumatic event). Depression and anxiety are known to be associated with traumatic life experiences.^29^ Likewise, the discriminant validity was proved with no significant correlation with the demographic variables that were judged to be unrelated to the number of traumatic events.

Exploratory factor analysis suggested a six factor-structure, which appears to fit the nature of traumatic events. Interestingly, non-specific, non-trauma-focused 'other very stressful event' and 'severe human suffering' were categorized with 'physical assault' (Factor 1: Physical assault/others). The interpersonal nature of physical violence, which possibly arises in relationships with partners or family members, may have caused 'human suffering' and the related other stressful events. There is also a possibility of 'human' translated in Korean may have a meaning of 'interpersonal'. Another point is that Factor 3 was a combination of seemingly different events (i.e., natural disaster, witnessing sudden death). A large scale trauma such as a natural disaster may have provided more chances for witnessing deaths.

The limitations of this study are as follows. First, we were unable to investigate the concurrent validity by comparing our measures with other established measures of PTEs, because the Korean language versions of the other instruments were not available.

Second, this study did not investigate the whole range of possible responses, but rather, this was limited to the direct exposure experiences. This was intended for the convenience of analysis and the internal validity, i.e., controlling the possible misapprehension and confusion over indirect exposures (e.g., media exposure). However, it should also be noted that having these multiple sources of exposure is an important merit of the LEC.

Third, the LEC excludes the aspects of personal reaction to PTEs that are necessary to fulfill criterion A of traumatic events. Consequently, when a person reports a listed event on the LEC, it does not mean that the person reacted with intense fear, horror, or terror. In this context, the LEC should be used as a screening tool for PTEs. Finally, the findings from this study are confined to psychiatric patients and so further research is needed to generalize the results to the general population.

Despite these weaknesses, this study confirmed the sound psychometric properties of the LEC-K. Therefore, the LEC-K can be easily administered for clinical populations as a screening tool for PTEs. This may in turn assist clinicians when they perform further trauma-related assessment and patient treatment.

## Figures and Tables

**TABLE 1:**
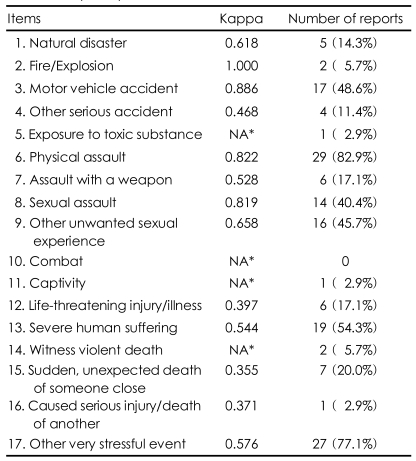
The four week test-retest reliability of the LEC-K in a subsample (n=35)

^*^Kappa was not computed because the variable was a constant. LEC-K: Life Events Checklist-Korean version, NA: not applicable

**TABLE 2:**
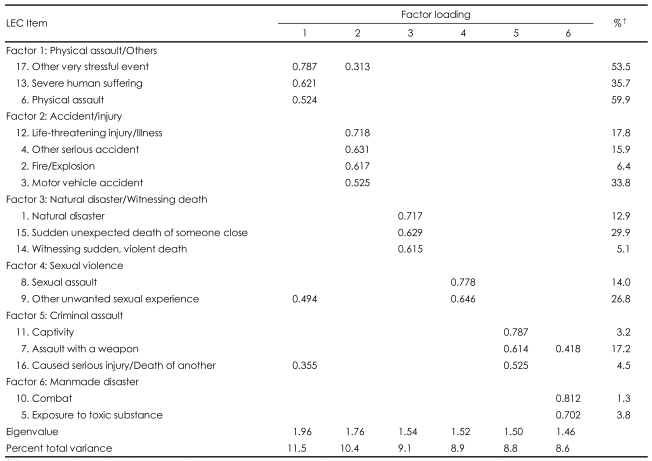
Principal component analysis^*^ of Life Events Checklist (LEC)-Korean version (n=157)

^*^Varimax rotation with Kaiser normalization, ^†^The percentage of endorsement in each item. Loadings smaller than 0.30 are not displayed
